# Supplementary material for: A small electron donor in cobalt complex electrolyte significantly improves efficiency in dye-sensitized solar cells
Source: Nat Commun. 2016 Dec 21;7:13934. doi: 10.1038/ncomms13934 (PMC5187592; doi:10.1038/ncomms13934)
Supplement: Supplementary Information — : Supplementary Figures, Supplementary Tables, Supplementary Notes, Supplementary References. [file ncomms13934-s1.pdf]

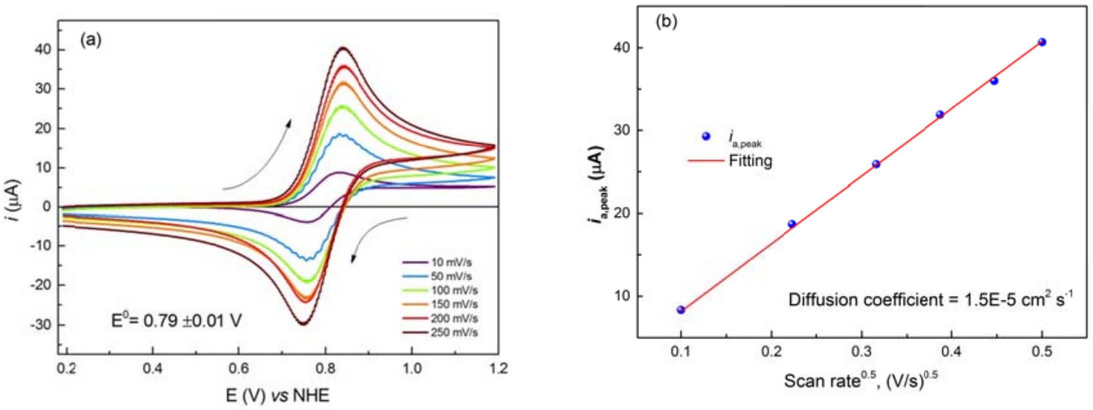

SUPPLEMENTARY FIGURE 1. (a) Cyclic voltammogram of 2.5 mM TPAA in 0.1 M $TBAPF_6$ in acetonitrile at different scan rates. (b) the diffusion coefficient was calculated by utilizing the relationship[1] between the scan rate and peak current: , where i_p is the anodic peak current, n is the number of transferred charge, A is the electrode surface area, C_0 is the concentration of the redox specie, D_0 is the diffusion coefficient and v is the scan rate. Symbol: original data, Red line: the fitting curve.

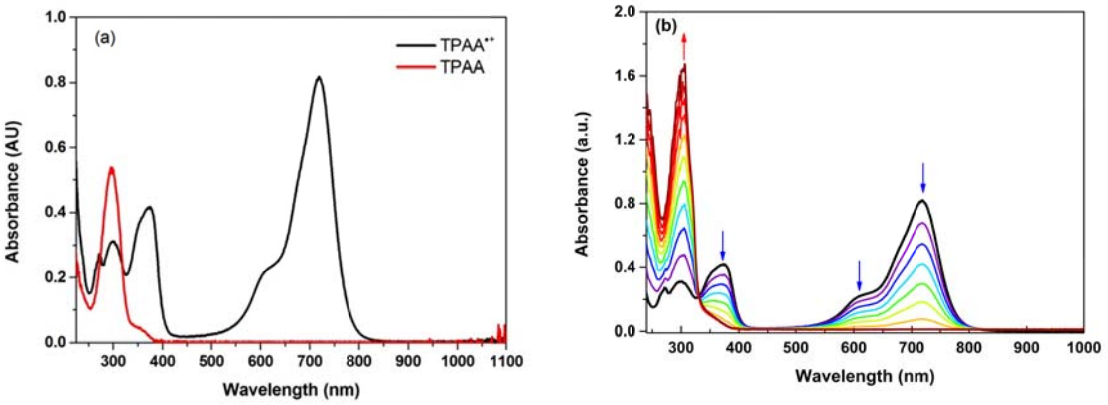

SUPPLEMENTARY FIGURE 2. (a) UV-vis absorption spectrum of TPAA (red line) and $TPAA^{\cdot+}$(dark line) (b) The absorbance changes of $TPAA^{\cdot+}$ with the titration of $[Co(bpy)_3]^{2+}$ by approx. 0.2 eq each addition. The blue arrow indicates the decreased absorption peaks at around 370 nm, 610 nm and 720 nm, while the red arrow indicates an increased absorption peak at 300 nm.

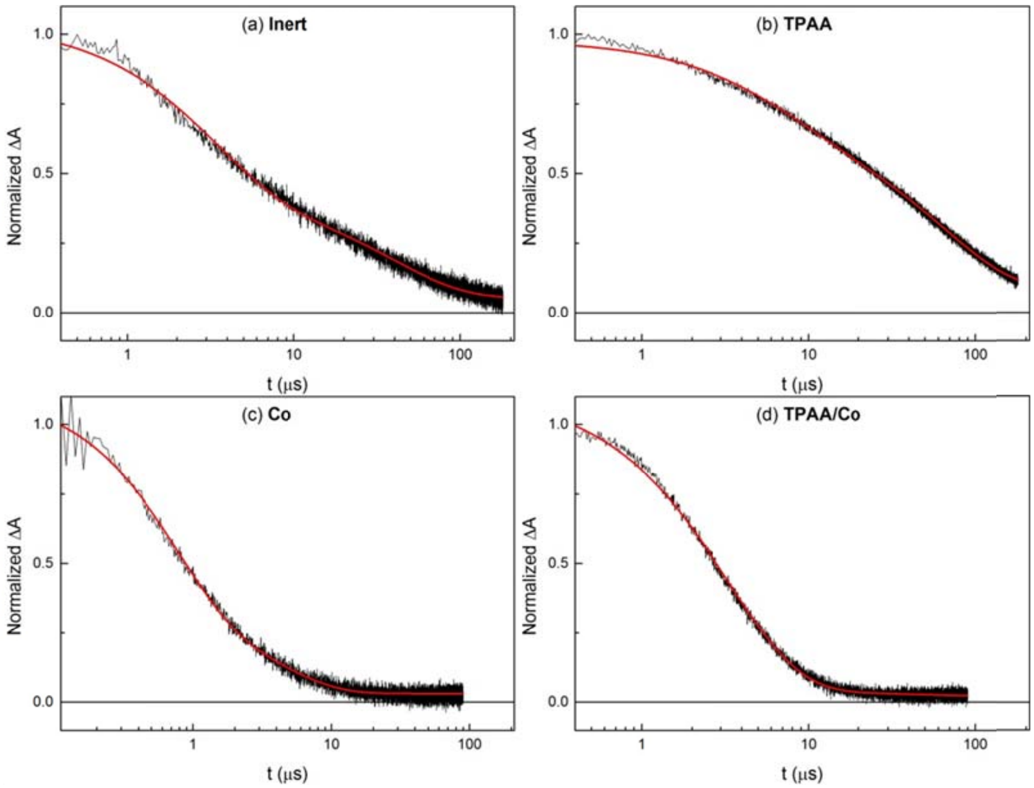

SUPPLEMENTARY FIGURE 3. Transient absorption kinetics of TiO_2 sensitized with **LEG4** dye under the condition of four different electrolytes (mentioned in the PIA figure caption). The laser intensity was controlled to be 0.15 to 0.25 mJ cm^{-2}.

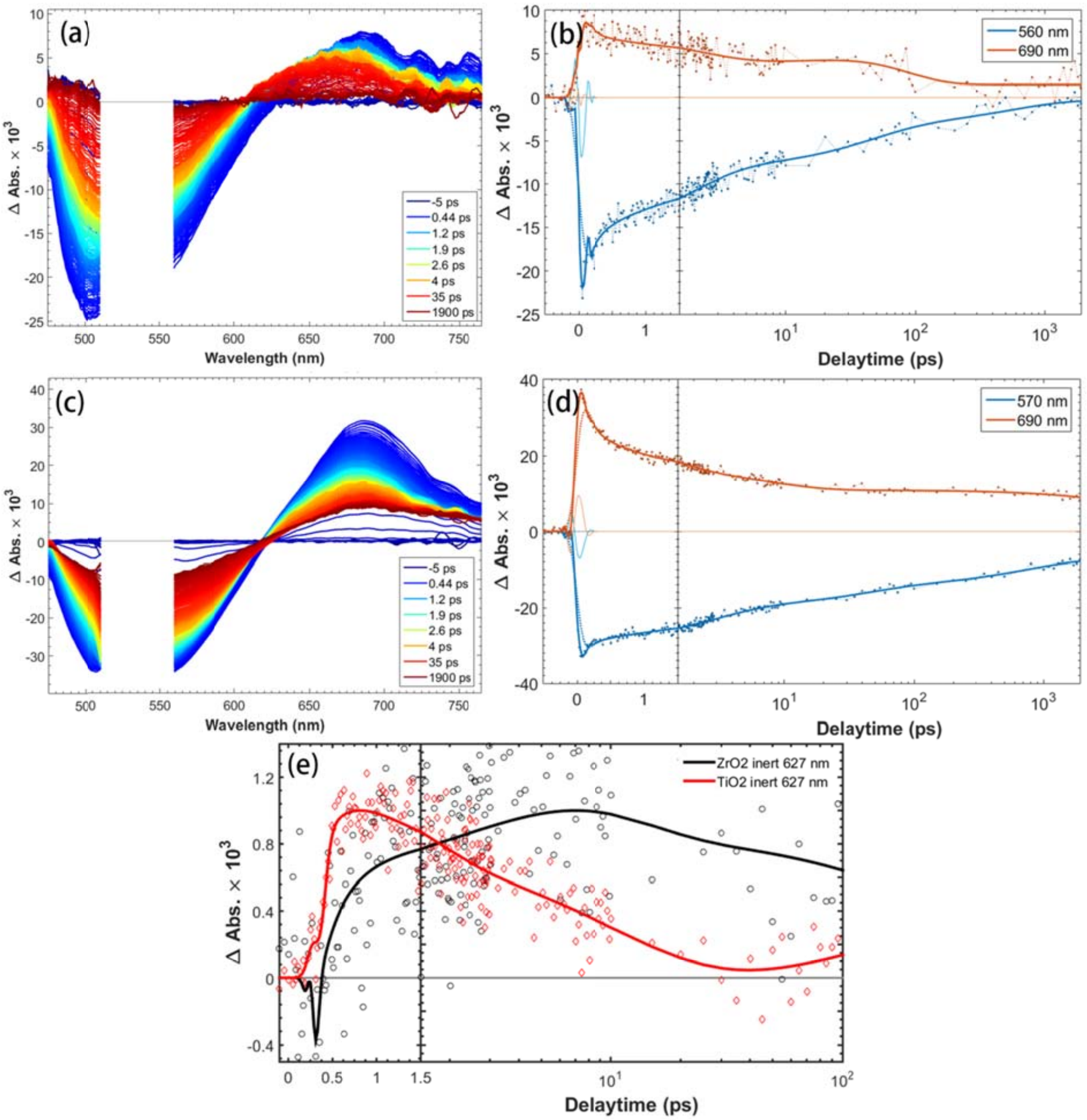

SUPPLEMENTARY FIGURE 4. (a) Ultrafast femtosecond (fs-TA) transient absorption spectra of LEG4/ZrO_2 in contact with the inert electrolyte (0.1 M $LiClO_4$ and 0.2 M TBP in propylene carbonate) (b) the normalized kinetic traces of LEG4/ZrO_2 probed at 560 and 690 nm. (c) fs-TA transient absorption spectra of LEG4/TiO_2 in contact with the same inert electrolyte (d) the normalized kinetic traces of LEG4/TiO_2 probed at 570 and 690 nm (e) Normalized kinetic traces of the change of absorbance close to the isosbestic point (around 627 nm) (O) LEG4/ZrO_2) (◊) LEG4/TiO_2 in presence of inert electrolyte.

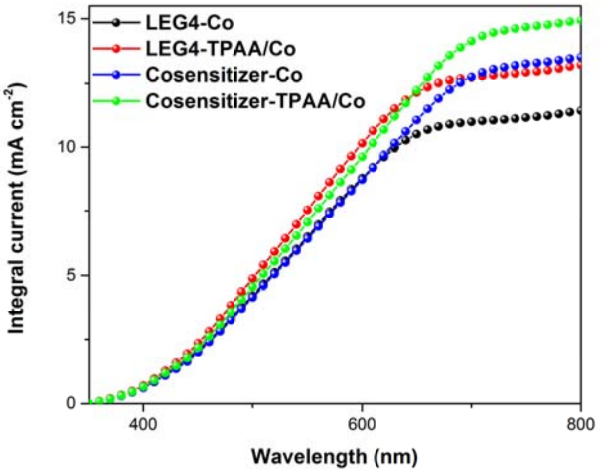

SUPPLEMENTARY FIGURE 5. The integral current from spectra of incident photon-to-current conversion efficiency (IPCE) for DSSCs based on LEG4 dye and co-sensitized (D35/Dyenamo Blue).

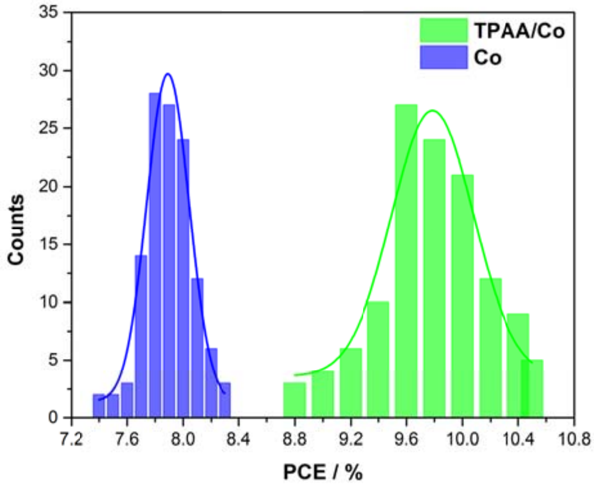

SUPPLEMENTARY FIGURE 6. Histogram of devices efficiencies based on co-sensitizer (D35/Dyenamo Blue) with **Co** (blue column) electrolyte and **TPAA/Co** (green column) electrolyte.

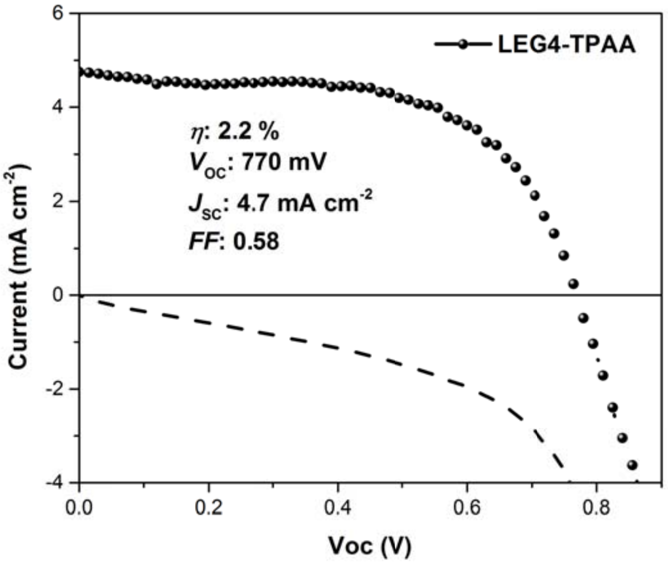

SUPPLEMENTARY FIGURE 7. *J-V* curves of DSSCs based on LEG4 as sensitizer and **TPAA** electrolyte. The electrolyte composition was 0.2 M TPAA, 0.1 M $LiClO_4$, 0.2 M 4-tert butylpyridine (TBP) and ~ 0.05 M $NOBF_4$ in acetonitrile. The sensitized-TiO_2 films and the preparation of the solar cells are the same as that described in the main text.

SUPPLEMENTARY TABLE 1. The rest potential of standard cobalt electrolyte with and without addition of 0.1 M TPAA.

	Voltage vs Ag/AgCl (1M LiCl in ethanol)	Voltage vs NHE
Standard cobalt	0.303 V	0.503 V
Standard cobalt+0.1 M TPAA	0.303 V	0.503 V

SUPPLEMENTARY TABLE 2: Summary of the rate and time constants calculated from the fitting

Sample	A_1	τ_1, μs	A_2	τ_2, μs	t, μs
**Inert**	0.62	3.2	0.38	39.4	8.3
**TPAA**	0.27	6.6	0.60	65.3	32.1
**Co**	0.68	0.7	0.29	4.1	1.2
**TPAA/Co**	0.98	3.3	0.02	51.8	3.5

SUPPLEMENTARY TABLE 3: Summary of time constants of the multiexponential fittings

	τ_1 (ps)	τ_2 (ps)	τ_3 (ps)	τ_4 (ps)
LEG4/ZrO_2	3.7/rise	36.5	856	∞
LEG4/TiO_2	0.76/rise	12.3	351	∞

SUPPLEMENTARY NOTE 1. We examined the rest potential of the electrolyte before and after addition of 0.1 M TPAA. Briefly, an electrode was assembled from a Pt wire in contact with the electrolyte in a plastic tube with a frit top allowing the free movement of ions. The potential difference between the assembled electrode and an Ag/AgCl (1M LiCl in ethanol) reference electrode was then measured in the supporting electrolytes (0.1 M $TBAPF_6$ in ACN), with use of the digit precision digital Keithley 2700 voltage meter. The reference electrode potential was then calibrated by Fc/Fc^+ in the same supporting electrolyte after measurements. The results are summarized in Supplementary Table 1. The accuracy of the measured potentials was estimated to be around 5 mV.

SUPPLEMENTARY NOTE 2. The normalized decay curves were fitted by a sum of two exponential functions (Supplementary Equation 1), from which logarithm averaged time constant was calculated according to Supplementary Equation 2.

$$\Delta \mathrm{A} = \mathrm{A}_0 + A_1 \exp(-\tau_1 t) + A_2 \exp(-\tau_2 t)$$ Supplementary Equation 1

$$\log(t) = \frac{A_1}{\mathrm{A}_1+\mathrm{A}_2}\log(\tau_1) + \frac{A_2}{\mathrm{A}_1+\mathrm{A}_2}\log(\tau_2)$$ Supplementary Equation 2

The weighted averages of lifetimes avoid overemphasizing the longer lifetime components, and give equal weight to the decadic time scale. The resulting time constant show great consistence with the inspect half-time in the figure and therefore used herein. The fitting data were summarized with Supplementary Table 2.

SUPPLEMENTARY NOTE 3. Ultrafast femtosecond (fs-TA) transient absorption spectroscopies were performed to investigate the charge transfer processes. Figure S4a shows the fs-TA spectra of LEG4 sensitized ZrO_2 film (LEG4/ZrO_2) in contact with 0.1 M $LiClO_4$ and 0.2 M TBP electrolyte in propylene carbonate at different delay time after laser excitation. Two main features were presented: a) a photo-induced transient ground state bleach at ~ 500 nm and b) a photo-induced excited state absorption in the range of 600 nm-760 nm. The vibrational relaxation of the excited state and presumably stimulated emission (~ 730 nm) are found to almost complete at initial 2 ps indicated by the stabilization of the isosbestic point. Decay of the difference spectrum at 690 nm shows anexcited state with a main characteristic lifetime 856 ps (Supplementary Figure 4b and Supplementary Table 3), in consistence with the absence of electron injection on ZrO_2 nanoparticle. In contrast, scarcely blue shift was noticed for the fs-TA spectra of LEG4-senstized TiO_2 (LEG4/TiO_2) in the same electrolyte (Supplementary Figure 4c). Instead, a slight red shift of the isosbestic point around 625 nm is found to be coupled with a gradually broadened spectrum. This is attributed to the ultrafast and efficient electron injection from the excited state LEG4 into TiO_2, even before the relaxation of the excited LEG4. The kinetic traces of the isosbestic point of the TA spectra in these two cases are shown in Supplementary Figure 4e. It is conceivable, therefore, that the electron injection occurs in a sub ps time scale, leading to the rapid rising of the isosbestic in the case of LEG4/TiO_2.

SUPPLEMENTARY REFERENCES:

1. Yang, W.; Vlachopoulos, N.; Hao, Y.; Hagfeldt, A.; Boschloo, G., Efficient dye regeneration at low driving force achieved in triphenylamine dye LEG4 and TEMPO redox mediator based dye-sensitized solar cells. *Phys. Chem. Chem. Phys* **2015,** *17* (24), 15868-15875.
